# Valorization of Low-Nitrogen, High-Organic-Load Shrimp Aquaculture Wastewater by *Dunaliella salina*: Pollutant Removal and High-Value-Biomass Production

**DOI:** 10.3390/microorganisms13071484

**Published:** 2025-06-26

**Authors:** Alvaro Barreto, Victor Manuel Luna-Pabello, Manuel Sacristán de Alva, Iveth Gabriela Palomino Albarrán, Martín Arenas, Gabriela Gaxiola

**Affiliations:** 1 Laboratorio de Microbiología Experimental, Departamento de Biología, Facultad de Química, Universidad Nacional Autónoma de México, Av. Universidad No. 3000, Col. Universidad, Nacional Autónoma de México C.U., Delegación Coyoacán, Ciudad de México C.P. 04510, Mexico; alvarobarreto09@gmail.com; 2 Unidad de Química Sisal, Facultad de Química, Universidad Nacional Autónoma de México Sisal, Sisal C.P. 97356, Yucatán, Mexico; m.sacristan@quimica.unam.mx; 3 Unidad Multidisciplinaria de Docencia e Investigación (UMDI) Sisal, Facultad de Ciencias, Universidad Nacional Autónoma de Mexico, Sisal C.P. 97356, Yucatán, Mexico; gabriela.palomino@ciencias.unam.mx (I.G.P.A.); mggc@ciencias.unam.mx (G.G.); 4 Departamento de Recursos del Mar, Centro de Investigación y de Estudios Avanzados del IPN (Cinvestav), Mérida C.P. 97310, Yucatán, Mexico; martin.arenas@cinvestav.mx

**Keywords:** shrimp aquaculture wastewater, chemical oxygen demand reduction, biofloc production systems, high-value biomass, polyunsaturated fatty acid, sustainable wastewater management

## Abstract

The rapid expansion of shrimp aquaculture has led to the generation of nutrient-rich effluents, which contribute to environmental degradation if inadequately managed. This study evaluated the potential of *Dunaliella salina* for the reuse of shrimp aquaculture wastewater (SAW) in biofloc production systems under varying dilution levels (0%, 25%, and 50%) and the simultaneous production of high-value biomass. Growth kinetics were modeled using a four-parameter logistic model, and nutrient removal, biochemical composition, and fatty acid profile were assessed. *D. salina* exhibited substantial growth in undiluted SAW, achieving over 80% removal of total nitrogen and reducing the organic load, as measured by a chemical oxygen demand reduction of more than 79%. In SAW treatments, the protein content ranged from 24.7% to 26.3%, while the lipid content reached up to 67.1% in a 25% SAW dilution. Chlorophyll *a* and total carotenoids were measured at 5.3–7 µg/mL and 4.1–5.7 µg/mL, respectively, in SAW treatments. The polyunsaturated fatty acid content in undiluted SAW was 34.5%, with α-linolenic acid (C18:3n3) and linoleic acid (C18:2n6) comprising 12% and 7.5%, respectively. This study demonstrates the ability of *D. salina* to valorize shrimp aquaculture wastewater in biofloc systems into lipid-rich, bioactive biomass, supporting its use in integrated aquaculture biotechnology systems for sustainable wastewater management and bioproduct generation.

## 1. Introduction

Shrimp aquaculture has become an important global industry owing to its significant economic impact and rapid growth. This sector has become a vital component of the global seafood supply chain, contributing substantially to global aquaculture production [1]. As the fastest-growing food production industry globally, aquaculture, including shrimp farming, is considered to have the greatest potential to meet the growing demand for seafood and to address overfishing concerns [2].

However, intensive and super-intensive farming practices in the shrimp aquaculture industry have led to increased wastewater generation [3]. Shrimp aquaculture wastewater (SAW) is characterized by high concentrations of ammonia, nitrite, and nitrates [4]. Additionally, wastewater contains high levels of carbon and organic matter, measured as the chemical oxygen demand (COD) [5]. The composition varies depending on farming practices, and the specific composition of shrimp farming wastewater can also differ [6]. In biofloc technology (BFT) systems dominated by heterotrophic bacterial assimilation, ammonium serves as the primary nitrogen source, while organic matter constitutes the principal carbon substrate [7]. Under heterotrophic-predominant BFT conditions, total nitrogen concentrations are typically reduced; however, elevated COD levels can be relatively high, primarily due to the accumulation of organic matter. Zhang et al. [8] reported that, in a conventional biofloc system, the average COD concentration is 229 mg/L in the middle-to-late culture stages. Nutrient concentrations in SAW can lead to eutrophication in coastal waters [9]. Furthermore, excess organic waste negatively affects oxygenation and alters essential biological processes, thereby affecting the aquatic ecosystems [10].

Interestingly, although wastewater poses environmental risks, it also presents opportunities for resource recovery and sustainable practices. For instance, the nutrient-rich wastewater can be used for cultivating microalgae which can help in nutrient removal and potentially produce valuable biomass [11,12].

Previous studies have demonstrated the potential of various microalgal species to treat aquaculture wastewater and to produce valuable biomass [13,14]. This dual functionality of microalgae is particularly significant in the context of sustainable aquaculture. Microalgae can effectively remove excess nutrients such as nitrogen and phosphorus from wastewater, thereby mitigating the environmental impact of aquaculture effluents [12]. Nutrient uptake by microalgae promotes algal biomass growth, which can be harvested for various applications. For example, the biomass produced can be converted into biofuels or animal feed [15,16].

*Dunaliella salina* Teodoresco, 1905, is primarily known for its ability to grow in saline environments and produce valuable bio-compounds like carotenoids, lipids, and proteins [17]. Although it is effective for removing carbon, nitrogen, and phosphorus from synthetic wastewater [18], specific studies quantifying its capacity to reduce organic matter, particularly chemical oxygen demand (COD), in aquaculture wastewater are scarce. Interestingly, other microalgae species have demonstrated significant organic matter removal capabilities for aquaculture wastewater treatment. For instance, *Parachlorella kessleri* showed COD removal of 94.4% in fishery wastewater [19].

However, there remains a gap in the literature regarding *D. salina* application for organic matter removal in shrimp aquaculture wastewater, particularly from biofloc production systems characterized by low nitrogen and high organic loads. Investigating the efficiency of *D. salina* in this context could provide insights into more sustainable and productive wastewater treatment methods.

This study aimed to evaluate the efficiency of *D. salina* in organic matter load removal from shrimp aquaculture wastewater and assess the production of high-value biomass. Specifically, proteins, lipids, fatty acids, and pigments, including kinetic models, have been assessed to obtain growth parameters and elucidate the growth dynamics of *D. salina* under aquaculture wastewater conditions.

## 2. Materials and Methods

### 2.1. Microalgae Collection and Molecular Identification

The microalgae *Dunaliella salina* was isolated from a water sample from Las Coloradas, Yucatán, Mexico (21°36′18″ N, 88°0′20″ W). All samples were stored in plastic bottles until their transfer to the laboratory at UMDI (Unidad Multidiciplinaria de Docencia e Investigación), UNAM (Universidad Nacional Autónoma de México) Sisal, Yucatán, México; then, the water was enriched with Guillard F/2 culture medium (GF2CM) [20] and incubated in 1 L Erlenmeyer flasks (Corning, Glendale, AZ, USA) before being placed in a single-cell nutrient plate containing 1.5% (*w*/*v*) agar. Colonies containing only one algal species were transferred to new GF2CM. DNA extraction was performed using the SDS method [21] and samples were stored at −20 °C. The oligonucleotides MA1 (5′-CGG GAT CCG TAG TCA TAT GCT TGT CTC-3′) and MA2 (5′-CGG AAT TCC TTC TGC AGG TTC ACC-3′), used to identify the microalga *Dunaliella salina*, were designed from 18S rDNA genes and were previously reported by Olmos et al. [22]. PCR reactions were carried out in a total volume of 25 µL, which included 50 ng of chromosomal DNA, 0.4 μM of each primer, 200 μM of each dNTP (Promega, Fitchburg, WI, USA), 0.6 × reaction buffer (Promega, Fitchburg, WI, USA), and 1.5 units of Taq DNA polymerase (Promega, Fitchburg, WI, USA) [23]. Amplification was performed in a thermocycler (Bio-Rad, Hercules, CA, USA) for 25 cycles, with each reaction having a melting temperature of 52 °C. A single cycle consisted of 1 min at 95 °C, another minute at 52 °C, and finished with 2 min at 72 °C [22]. The PCR products were subjected to 2% agarose gel electrophoresis for analysis, and the purified DNA was recovered for sequencing. The obtained sequence was used for identification via a BLAST (v2.15.0) search at the National Center for Biotechnology Information (NCBI) (http://www.ncbi.nlm.nih.gov/BLAST/, accessed on 3 February 2025).

### 2.2. Aquaculture Wastewater Collection

Shrimp aquaculture wastewater (SAW) was collected after its discharge from the shrimp culture units from UMDI-Sisal, UNAM in Yucatán, Mexico. Shrimp management was described by Eden et al. [24]. Before inoculation with microalgae cells, raw wastewater was autoclaved at 121 °C under 138 kPa for 15 min. This sterilization process eliminated endogenous microbial competitors and pathogens (e.g., bacteria, protozoa), served our experimental purposes, isolated the specific contributions of *Dunaliella salina* to nutrient removal and biomass production, and enabled the controlled assessment of potential contaminant effects by establishing a sterile baseline. Consequently, observed changes in nutrients and biomass were exclusively attributable to *D. salina*, providing an unambiguous evaluation of its bioremediation potential.

### 2.3. Experimental Desing

Initial stock cultures were maintained in GF2CM, with an initial inoculum density of 2.5 × 10^5^ cells/mL. Incubation was conducted in 1 L Erlenmeyer flasks containing the following proportions of marine water and aquaculture wastewater: undiluted SAW was labeled “0%” at 25% dilution with marine water (75% SAW) labeled “25%” and a 50% dilution (half marine water, half SAW) was labeled “50%”. The GF2CM treatment was employed as a reference, replicating the autotrophic conditions under which the initial stock culture was maintained. This treatment established a baseline for comparative analysis against wastewater treatments, which operated under mixotrophic conditions due to the presence of organic matter in the medium. All treatments were performed in triplicate. It is important to note that the dilutions were made using sterile seawater without the addition of GF2CM components. Microalgal cultures were maintained with continuous aeration at a flow rate of 0.5 L/L/min; salinity of 38 ‰; temperature of 24 ± 1 °C; and illumination from cool white fluorescent lamps at 350 µmol photons/m^2^/s under continuous light conditions.

### 2.4. Determination of Dunaliella salina Growth

Algal cell density was counted every 24 h using a Neubauer hemocytometer under a microscope (Velab, Ciudad de Mexico, Mexico).

Microalgal growth was performed using a four-parameter logistic model, as shown below in Equation (1):

Cell_ij_ = A + B_i_/1 + exp[(xmid − time_ij_)/exp(scal)] (1)

where Cell is the cell density of each flask; i is the time_ij_ in days (j range 1 at 8); *A* is the asymptote on the left side; B is the asymptote on the right side; xmid is the value of time that the cell density is midway between the asymptotes; and scal is the slope at the inflection point at the midrange.

Growth data were fitted to the logistic (four-parameter) model using the R packages “nlraa” and “nlme” [25,26] in the R statistical language and environment, version 4.2.2 [27]. To determine the best-fitting model for the growth data, the Akaike Information Criterion (AIC) and Bayesian Information Criterion (BIC) were used. Both criteria utilize the likelihood value to measure the fit and number of parameters to assess model complexity [28].

In the next step, the temporal variation in growth rates was assessed using a nonlinear growth model and specific growth rate (µ) was calculated over time. The approach described by Paine et al. [29] was used to determine and compare specific growth rates among treatments.

### 2.5. Nitrogen and Chemical Oxygen Demand Removal

Pollutant removal was monitored every 48 h. Briefly, 10 mL of sample was collected from the culture flask and filtered through a 0.45 μm filter (cellulose nitrate filter, Whatman 7141–004, 47 mm, USA). These samples were analyzed for nitrates, nitrites, phosphorus, and total ammonia nitrogen using spectrophotometry [30,31] with a microplate reader (Synergy HT, BioTek, Winooski, VT, USA).

Organic matter load measured as chemical oxygen demand (COD) was determined using the closed reflux colorimetric method described by Vyrides and Stuckey [32]. To estimate the total organic carbon (TOC) from chemical oxygen demand (COD) measurements in wastewater, a COD:TOC ratio of 3, as reported by Dubber and Gray [33], was utilized.

The percentage of pollutant removal was calculated using the following equation:

R = Si − Sf/Si × 100

where *R* is the percentage of pollutant removal; *Si* is the initial concentration of the pollutant; and *Sf* is the concentration remaining at the end of the cycle.

### 2.6. Biochemical Biomass Composition

Protein determination was measured using the microbiuret method [34], with modifications proposed by Chen and Vaidyanathan [35]. In brief, proteins were extracted using an alkaline method. A 2 mL aliquot of the sample was centrifuged at 12,000× *g* for 2 min, and 1 mL of 0.5 N NaOH was added to the pellet. The mixture was then heated at 80 °C for 10 min. This alkaline extraction process was repeated three times. The protein content in the extracts was measured using a rapid and sensitive micro-biuret method. A 0.05 mL aliquot of copper sulfate solution (0.21% CuSO_4_·5H_2_O in 30% NaOH) was added to 0.1 mL of the samples, and absorption was measured at 310 nm. Bovine serum albumin was used as the standard for calibration.

Lipid extraction from 100 mg of lyophilized microalgae was conducted using a modified version of Folch’s extraction method [36]. The extraction involved an ultrasound-assisted process using a dichloromethane/methanol solution in a 2:1 volume ratio [37]. The lipid extracts were evaporated, and total lipid content was determined by gravimetric analysis.

Chlorophyll and total carotenoid content were measured using a spectrophotometric method following extraction with 90% acetone. Chlorophyll levels were calculated using the equations provided by Humphrey [38], while total carotenoid content was determined using the equation from Strickland and Parsons [39].

### 2.7. Fatty Acid Profile Determination

The determination of fatty acid methyl esters (FAMEs) was carried out using gas chromatography (Perkin Elmer Clarus 500, PekinElmer, Shelton, CT, USA). Lipid extracts from the microalgae were first trans-esterified. Thus, lipids were extracted from 100 mg of lyophilized microalgae. They were then mixed with 2 mL of methanol, pH-adjusted to 1–2, heated at 80 °C for one hour. After cooling, 2 mL of hexane and 1 mL of distilled water were added to the mixture. The hexane layer, containing FAMEs, was separated and dried over anhydrous sodium sulfate. The resulting FAMEs were analyzed using a gas chromatograph equipped with a flame ionization detector and a capillary column (Phenomenex, Carlsbad, CA, USA. The column temperature was programmed to rise from 120 to 250 °C at a rate of 10 °C per minute, and the injection volume was set at 1 µL. Identification and quantification of the individual fatty acids were achieved by comparing the retention times and peak areas with those of known standards [40].

### 2.8. Antioxidant Enzyme

Superoxide dismutase (SOD, EC 1.15.1.1) was determined using the method described by Flohé and Ötting [41]. One unit of SOD was defined as the amount of enzyme required to inhibit the cytochrome c reduction rate by 50%. Glutathione peroxidase (GPx, EC 1.11.1.9) was measured using Paglia and Valentine’s [42] protocol, as modified by Marie et al. [43]. Activity was measured using a NADPH standard curve. Catalase (CAT, EC 1.11.1.6) was measured using Hadwan and Najim’s [44] method, based on the reaction of undecomposed hydrogen peroxide with ammonium molybdate to produce a yellowish color with maximum absorbance at 374 nm. Ascorbate peroxidase (APX, EC 1.11.1.11) was determined with the method described by Nakano and Asada [45], recording the decrease in optical density at 290 nm due to ascorbic acid. Enzyme activity was expressed by calculating the decrease in ascorbic acid content compared to a standard curve drawn from known concentrations of ascorbic acid.

### 2.9. Statistical Analysis

All statistical analyses and graphical visualizations were performed using R software version 4.2.2 [27]. Data are presented as the mean ± standard error. Microalgal growth was performed with a nonlinear mixed effects model using a four-parameter logistic model.

To assess model validity, Pearson’s residuals were examined in three ways: against each explanatory variable and fitted values, and by plotting observed data versus fitted values. Significance was assigned at *p* < 0.05. When significant differences were observed, post hoc tests were performed with Tukey’s method for data adjustment from the “emmeans” and “multcomp” packages [46,47]. Biochemical biomass component and antioxidant enzyme activity were evaluated with univariate analysis of variance.

Principal Component Analysis (PCA) was utilized to assess alterations in fatty acid profiles of *Dunaliella salina* microalgae grown in shrimp aquaculture wastewater and GF2CM. Prior to calculating the resemblance matrices of Euclidean distances between samples, multivariate datasets of the response variables were normalized. A one-way permutational multivariate analysis of variance was performed with 9999 permutations of residuals under a reduced model [48].

## 3. Results

### 3.1. Microalgal Growth

**Figure 1:**
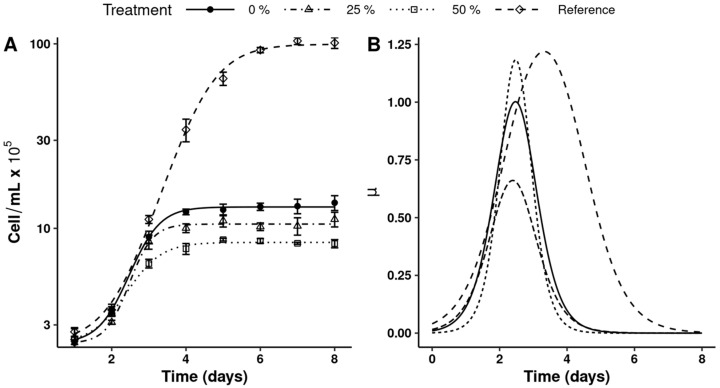
*Dunaliella salina* growth data in aquaculture wastewater for (**A**) cell density; (**B**) specific growth rate (µ) derivation of nonlinear model. In (**A**), the point and error bar indicate mean ± standard error and the lines indicate the four-parameter logistic model prediction. In (**A**,**B**), the difference in line type indicates treatments with 0, 25, and 50% in aquaculture wastewater dilution, respectively. The reference was grown in Guillard F2 culture medium.

The experimental growth data of *Dunaliella salina* microalgae, cultivated in different concentrations of shrimp aquaculture wastewater (SAW), are shown in Figure 1A, with the estimated parameters presented in Table 1. Significant differences were observed on the right-side asymptote (parameter B), representing the maximum cell density growth, across all treatments. Among the treatments with SAW, the treatment without dilution (0%) showed the best growth with a 23.79% increase compared to the 25% dilution treatment. In contrast, the reference treatment using GF2CM exhibited a seven-fold higher growth compared to SAW treatments.

The time at which cell density was halfway between the asymptotes (xmid) was similar among SAW treatments. This parameter also indicates the time at which the maximum absolute growth rate (derivative of cell density with respect to time) is reached. The slope parameter at the midrange (scal) was similar between the reference treatment and the treatment with the highest dilution (50%). However, the treatments with higher SAW (0% and 25%) concentrations exhibited a lower slope.

Figure 1B displays the temporal variation in specific growth rate (SGR) as determined by the nonlinear model. In the present study, the maximal SGR for the 25 and 0% treatments was reached at 2.5 days, occurring one day earlier than in the GF2CM, and was 3 to 22% lower in magnitude, respectively. The lowest SGR was observed in the treatment with SAW 50% dilution.

The growth patterns of *Dunaliella salina* in GF2CM and SAW differed significantly, reflecting the distinct trophic modes of the media. While GF2CM supported higher cell densities and specific growth rates, SAW, as a mixotrophic culture, exhibited lower growth performance. These differences may be attributed to an imbalance in the carbon-to-nitrogen (C:N) ratio in SAW or the presence of inhibitory compounds. Despite these conditions, *D. salina* demonstrated the capacity to grow in undiluted SAW, underscoring its adaptability to nutrient-stressed, organic-rich environments.

### 3.2. Nitrogen Removal and Chemical Oxygen Demand Reduction

The SAW used in the present study contained nitrogen at the following concentrations: nitrate 1.69 mg/L, nitrite 0.63 mg/L, total ammonia nitrogen (TAN) 0.81 mg/L, chemical oxygen demand (COD) 458.1 mg O_2_/L, and total nitrogen 3.1 mg/L, and the phosphate levels were undetectable with the methodology used in this study. Considering the total organic carbon (TOC) estimated from the COD [33], it is possible to propose an approximate carbon-to-nitrogen (C:N) ratio of 44:1 in SAW. The nutrient concentrations observed in the present study are similar to those found in biofloc technology but are generally lower in nitrate concentration compared to the recirculation system, as shown in Table 2.

**Figure 2:**
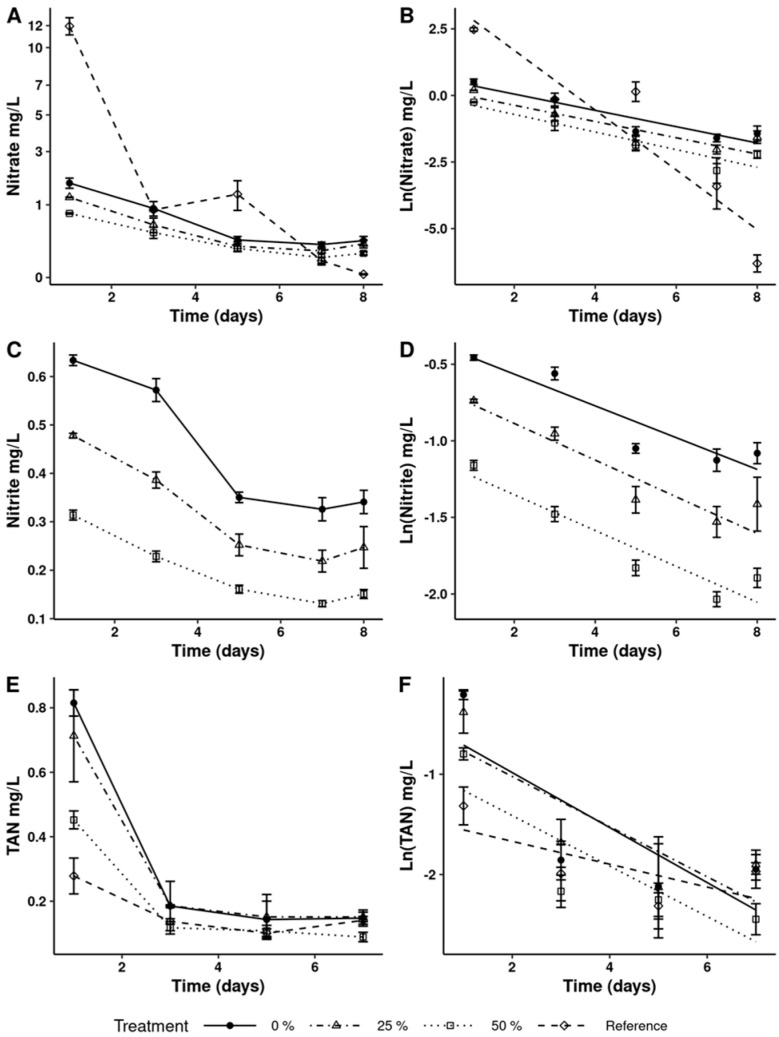
Concentration of nitrate (**A**) and nitrite (**C**); total ammonia nitrogen (**E**); and semilog transformation (**B**,**D**,**F**) during *Dunaliella salina* cultured in wastewater.

**Figure 3:**
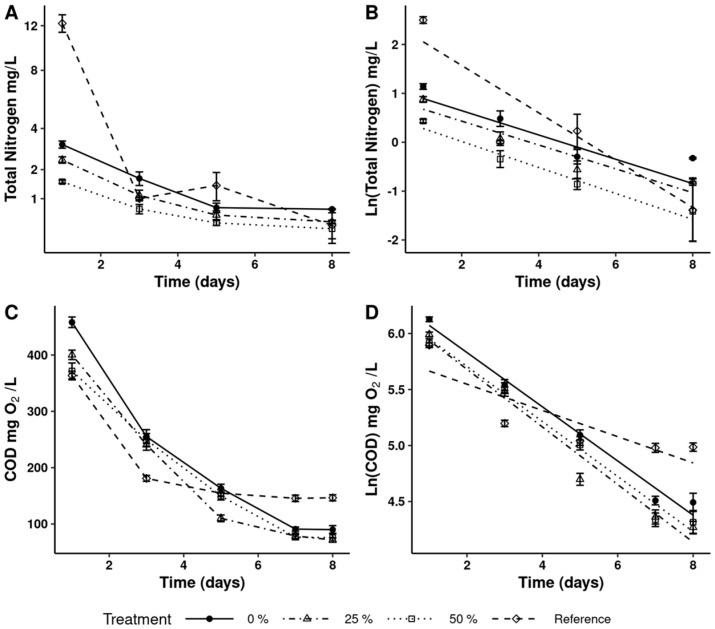
Concentration of total nitrogen (**A**); chemical oxygen demand (**C**); and semilog transformation (**B**,**D**) during *Dunaliella salina* cultured in wastewater.

Figure 2 shows a decrease in nitrate, nitrite, and TAN concentrations in both SAW and GF2CM during the culture period. The decreases in total nitrogen and COD are shown in Figure 3.

The results presented in Table 3 show nutrient removal efficiencies higher than 80% for nitrate and TAN, except for the treatment with a 25% dilution of SAW, where the removal efficiency was 76%. Nitrite removal was close to 50% for all treatments with SAW.

### 3.3. Biochemical Biomass Composition and Antioxidant Enzyme System of Dunaliella salina

The protein and lipid results in the present study are shown in Table 4. SAW treatments exhibited comparable protein content, whereas the GF2CM reference showed a significantly higher protein concentration (*p* < 0.05). Chlorophyll *a* and total carotenoid levels were significantly reduced in SAW treatments relative to the GF2CM reference (*p* < 0.05). Antioxidant enzyme activities (superoxide dismutase (SOD), glutathione peroxidase (GPx), and catalase (CAT)) did not differ significantly across treatments (Table 5) (*p* > 0.05). In contrast, ascorbate peroxidase (APX) activity was markedly elevated in the GF2CM reference compared to the SAW groups (*p* < 0.05).

Table 6 shows the fatty acid (FA) composition of *Dunaliella salina* microalgae cultivated in undiluted aquaculture wastewater (0% SAW), and in 25- and 50%-diluted SAW; the reference was grown in GF2CM. The highest saturated fatty acids (SFAs) were obtained in 50%-diluted SAW (*p* < 0.05). In all treatments, palmitic (C16:0) and oleic (C18:1n9) acids were the FAs that were at a higher concentration. The lowest value of myristotelic (14:1), palmitoleic (16:1), and eicosenoic (20:1n9) acids were found in the undiluted SAW and reference (*p* < 0.05). However, the amounts of monounsaturated fatty acids (MUFAs) were similar among all treatments (*p* > 0.05). The reference and undiluted SAW presented more α-linolenic acid (18:3n3) than the other treatments (*p* < 0.05). Eicosatrienoic (20:3n3), ecosadienoic (20:2n6), and arachidonic (20:4n6) acids were not detected in 25%- and 50%-diluted SAW. The lowest level of polyunsaturated fatty acids (PUFAs) was determined at a SAW dilution of 50% (*p* < 0.05).

**Figure 4:**
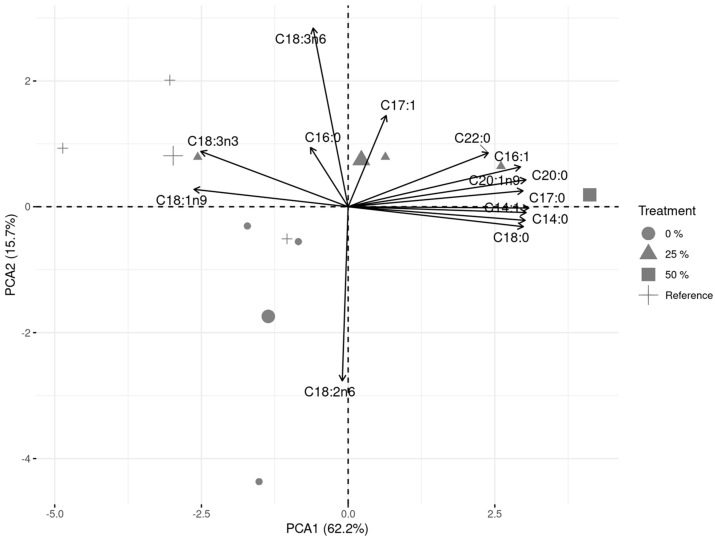
The Principal Component Analysis (PCA) applied to the fatty acid (FA) composition of *Dunaliella salina* microalgae cultivated in undiluted aquaculture wastewater (AWW) (0% AWW) and 25- and 50%-diluted AWW; the reference was grown in Guillard F2 culture medium.

**Figure A1:**
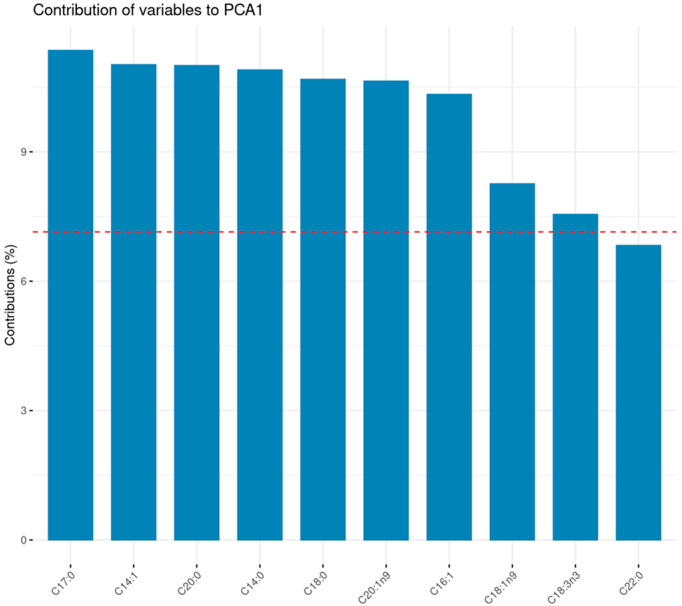
Top 10 variables that contributed the most to the horizontal ordination in the Principal Component Analysis applied to the fatty acid composition of *Dunaliella salina* microalgae.

**Figure A2:**
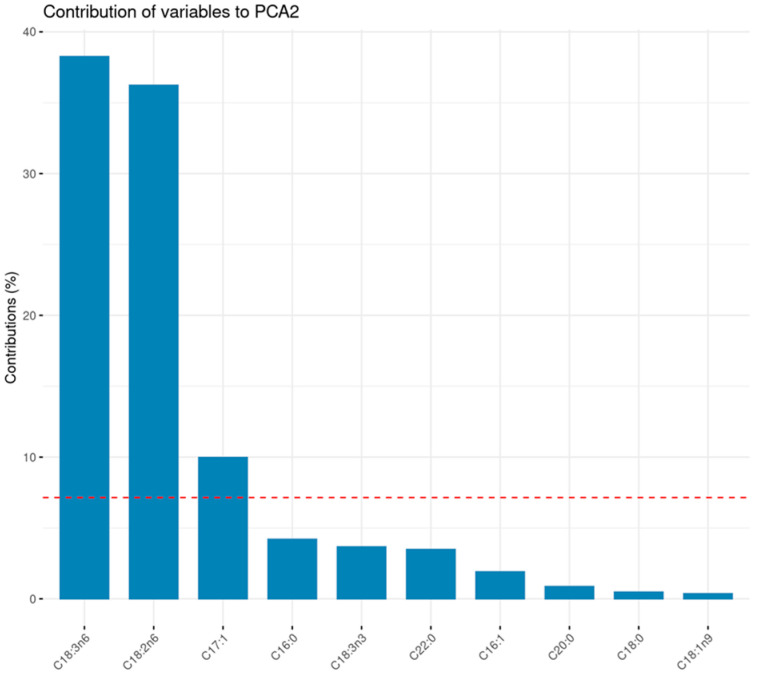
Top 10 variables that contributed the most to the vertical ordination in the Principal Component Analysis applied to the fatty acid composition of *Dunaliella salina* microalgae.

In the Principal Component Analysis (PCA) applied to *D. salina* FA composition in this study, approximately 78% of the total variation was explained in the first and second principal components (Figure 4). The ordination in PCA1 (horizontal) of FAs in *D. salina* showed that α-linolenic acid was the principal PUFA that contributed most to separating the reference and undiluted SAW, as well as MUFAs like oleic and eicosenoic acids (Figure A1). On the other hand, PCA2 (vertical) showed that undiluted SAW separation was mostly due to linoleic acid (C18:2n6); the other treatments’ separation was mostly due to γ-linolenic acid (C18:3n6) (Figure A2).

## 4. Discussion

### 4.1. Dunaliella salina Growth in Shrimp Aquaculture Wastewater

In the current study, the treatment without dilution demonstrated the highest growth out of the SAW treatments. The organic matter present in the wastewater facilitates the mixotrophic growth of microalgae, allowing them to utilize both light and organic compounds for their development. This was particularly evident in *Chlorella* sp., where an increase in the initial organic carbon/nitrogen ratio of wastewater resulted in an enhanced growth rate [56]. Chandra et al. [57] investigated the impact of organic carbon supplementation, specifically with 500 mg/L of glucose, on the growth and lipid production of microalgae under nutrient stress conditions. That study suggests that the presence of organic matter under such stress conditions can significantly enhance both the growth and lipid production in microalgae. These findings differ from the results of the present study, where the reference culture under autotrophic conditions exhibited superior growth compared to the mixotrophic culture in SAW. A plausible explanation for this growth pattern in *D. salina* is that, under nitrogen-limited conditions or elevated carbon-to-nitrogen (C:N) ratios, mixotrophic cultures may experience reduced growth relative to autotrophic cultures supplied with sufficient nitrogen. This behavior has been similarly reported for *Chlorella vulgaris* by Adesanaya et al. [58].

The primary advantage of using *Dunaliella salina* for water reuse is its high tolerance to varying nutrient concentrations and its ability to thrive in harsh conditions [13], such as those found in SAW. Previous works have reported similar findings regarding *Dunaliella* sp. growth and the kinetics parameters in various wastewater types. For instance, Malibari et al. [51] demonstrated that *Dunaliella* sp. could effectively be used in shrimp farm wastewater to support microalgal growth, showcasing its potential in nutrient removal. In another work, Wu et al. [59] highlighted *Dunaliella tertiolecta*’s ability to grow in food waste leachate, achieving significant biomass yields (0.5 g/L/day) and pollutant removal (>80%). These works align with our findings, reinforcing the viability of *D. salina* for SAW treatment applications.

### 4.2. Pollutant Removal

The nutrient concentrations observed in the present study were consistent with those reported in biofloc technology (BFT) systems [54,60]. Manipulation of the carbon-to-nitrogen ratio in BFT systems promotes the growth of bacterial communities that convert toxic nitrogenous wastes into microbial proteins [61]. This process effectively reduces ammonia, nitrite, and nitrate concentrations in culture water [62]. However, the accumulation of organic matter in BFT systems typically results in higher chemical oxygen demand (COD) [19].

The results of nutrient removal efficiencies were similar to those reported by Mirzaei et al. [63], who observed a 75% reduction in nitrogenous components with *Dunaliella salina* cultivated in salmon trout pond wastewater. However, the removal efficiency reported by Shiri et al. [64] was higher than that reported in the present study, since they used a fiber polyacrylonitrile–graphene oxide membrane bioreactor for shrimp wastewater treatment.

Sacristán et al. [18] reported a nitrogen removal efficiency higher than 90% in synthetic wastewater. A plausible explanation for this high removal efficiency is that the initial nitrogen concentration was higher than that used in our study. The results obtained with *Neochloris oleoabundans* indicate that nitrogen removal efficiency decreases with decreasing nitrogen concentration [65].

*D. salina* can grow under mixotrophic cultivation [66]. COD represents the total organic load in wastewater, including both dissolved and suspended matter [67]. In wastewater treatment, achieving higher COD removal is desirable, since it results in lower residual organic content. COD removal efficiency increased in the SAW treatments (>79%) compared to the reference (59%), indicating that *D. salina* utilized the carbon in the SAW of the BFT system for growth. These results are consistent with those reported by Daneshvar et al. [50] for *C. vulgaris*. In contrast, COD removal for *Tetraselmis* sp. was reported at 61%, suggesting that this microalga primarily utilized nitrogenous compounds in the aquaculture effluent [37].

The carbon-to-nitrogen (C:N) ratio plays a significant role in the effectiveness of microalgae in removing pollutants. Xu et al. [68] investigated influent C:N ratios of 2.5:1, 5:1, and 10:1 in the *Ganoderma lucidum* strain. The most efficient pollutant removal occurred at a C:N ratio of 5:1, which yielded substantially better results than the other ratios. This ratio achieved removal efficiencies of 71.89% for nitrogen, 69.34% for phosphorus, and 77.64% for COD.

The impact of elevated C:N ratios on nutrient removal efficacy can be explained through stoichiometric alignment with microalgal biomass composition such as C_106_H_263_O_110_N_16_P_1_, as proposed by Redfield [69]. When the C:N ratio of wastewater exceeds the optimal assimilation ratio (~6–10 mol C/mol N based on their elemental formula), nitrogen becomes growth-limiting. This imbalance forces microalgae to reduce nitrogen’s assimilation capacity due to N scarcity.

### 4.3. Biochemical Composition of Dunaliella salina Biomass

The protein and lipid results in the present study are consistent with those reported by Fabregas et al. [70], who found that protein content in microalgae increases with higher nitrogen concentrations in the medium. Fabregas et al. [70] showed that nitrogen levels below 2 mg/L were considered deficient for *Dunaliella tertiolecta*. The low nitrogen content of SAW treatment contributed to reduced protein content. Nitrogen starvation is known to decrease protein levels, a trend also observed in *D. salina* [71].

Nitrogen and phosphorus deprivation are known to increase lipid content and productivity in microalgae [72]. Our findings align with previous studies on *D. salina*, *Scenedesmus obliquus*, and *Chlorella sorokiniana* [13,73]. Maltsev et al. [74] analyzed nitrogen and phosphorus concentrations in nutrient media across various taxonomic and ecological groups of microalgae, identifying phosphorus levels below 0.019 mg/L as limiting. In the present study, the concentration of phosphorus was not detected, suggesting that the SAW used could be considered limiting.

A high carbon-to-nitrogen (C:N) ratio can significantly alter microalgal metabolic allocation by limiting nitrogen-dependent processes such as protein synthesis while promoting the accumulation of carbon-rich storage compounds, including lipids and carbohydrates. This metabolic shift can be interpreted in the context of the microalgal elemental stoichiometric formula, typically approximated as C_106_H_263_O_110_N_16_P_1_ [69]. Under conditions where nitrogen availability falls below the cellular demand implied by this ratio, excess assimilated carbon is diverted toward the synthesis of storage lipids and carbohydrates [57], resulting in reduced protein content and altered biochemical composition, as observed in this study.

The results for chlorophyll *a* and *b*, as well as total carotenoid content could be attributed to reduced nitrogen levels causing chlorophyll breakdown, which altered photosynthetic activities due to a reduction in electron acceptors, such as NADP+ [75]. However, when compared to fish wastewater and stickwater, chlorophyll *a* content was similar (Table 5). Carotenoid content can be enhanced in cultivation media with nitrogen limitation [71]. In contrast, our results show higher total carotenoid levels in the reference (nitrogen-replete) treatment. Nagappan and Kumar [76] reported that *Chlorella minutissima*, *D. tertiolecta*, and *D. salina* exhibited a reduction in carotenoid content under nitrogen limitation, while *Desmodesmus* sp. showed no change. Carotenoids are key components of endogenous non-enzymatic antioxidants that neutralize free radicals [77]. Our results are in accordance with a previous study on *Nephroselmis* sp., showing decreased peroxyl radical scavenging activity and total carotenoid content under nitrogen limitation or starvation conditions [78]. Therefore, our hypothesis is that in culture media with limited nitrogen concentrations, carotenoids play a more crucial role than enzymatic antioxidant systems.

### 4.4. Fatty Acid Composition of Dunaliella salina Cultivated in Shrimp Aquaculture Wastewater

The most prevalent saturated fatty acid in the present study was palmitic acid. Our results are consistent with those described by Fakhri et al. [79]. Conversely, Malibari et al. [51] reported a higher concentration of palmitic acid in *Dunaliella* sp. cultivated in F2 medium compared to shrimp farm wastewater. Malibari suggested that shrimp farm wastewater inhibits C16:0 elongase. The present study showed no evidence to suggest the presence of an inhibitory factor for C16:0 elongase in SAW. The highest amount of SFAs was measured in 50%-diluted SAW, which could be attributed to variations in nitrogen content among the treatments. Our findings align with those reported for *Desmodesmus* sp., *Chlorella* sp., *Dunaliella salina*, and *Dunaliella tertiolecta* [70].

The fatty acid (FA) composition of polyunsaturated fatty acids in undiluted SAW was similar to the reference. This result aligns with those reported for *Dunaliella* sp. by Malibari et al. [51] and Fakhri et al. [79]. The quality of FA from the treatment without dilution was higher than that reported for wastewater from shrimp farms, stickwater from a kilka fishmeal plant, and food waste leachate (Table 7).

Under nitrogen-limited, carbon-rich conditions, microalgae typically redirect assimilated carbon toward lipid biosynthesis, with a concomitant increase in saturated and monounsaturated fatty acids and a reduction in polyunsaturated fatty acids (PUFAs) [56]. This metabolic shift has been reported in *Chlorella vulgaris* and *Nannochloropsis oculate* under nitrogen starvation [80]. Similar trends were observed in the present study, with elevated levels of palmitic and oleic acids and a relative decline in PUFAs in SAW treatments with 50% dilution compared to the reference with autotrophic cultivation.

**Table 7:** Fatty acid (% of total fatty acids) profile of *Dunaliella* sp genus using wastewater as nutrient.

Fatty Acid	This Study’s SAW *	*Dunaliella* sp. [51]	*Dunaliella* sp. [79]	*Dunaliella tertiolecta* [59]	*Dunaliella salina* [81]
SFA					
C10:0			0.71		
C12:0	1.58		0.17		
C14:0	1.77	0.9	1.25	1.17	4.21
C15:0	1.57		0.89		0.58
C16:0	18.07	31.6	40.55	30.24	33.59
C17:0	1.23		1.37		0.36
C18:0	2.36	3.8	2.61	4.15	1.29
C20:0	2.06		0.71	1.53	
C22:0	2.31		2.9		0.1
MUFA					
C14:1	1.79	1.9	2.955	0.42	0.5
C16:1	1.79	4.7	5.86		5.12
C18:1	17.63	17	7.215	23.48	10.33
C20:1	1.67		2.9		0.04
C22:1					0.25
C24:1			6.98		0.06
PUFA					
C18:2	7.51	5.5	2.46	11.60	11.24
C18:3n3	12.03	13	1	7.62	3.9
C20:3n3	1.58		1.635		
C20:5		4.6	10.93		0.1
C22:2					0.05
C22:6			3.275		
ΣSFA	32.88	36.3	54.79	37.51	40.47
ΣMUFA	26.79	32	25.91	43.12	28.31
ΣPUFA	34.45	29.2	19.3	18.86	22.6

SFAs, saturated fatty acids; MUFAs, monounsaturated fatty acids; PUFAs, polyunsaturated fatty acids. * *Dunaliella salina* cultured in undiluted aquaculture wastewater (0% SAW).

## 5. Conclusions

This study demonstrated that *Dunaliella salina* can effectively utilize shrimp aquaculture wastewater (SAW) derived from biofloc systems, achieving significant pollutant removal and producing valuable biomass under mixotrophic conditions. The undiluted SAW treatment supported the highest microalgal growth among wastewater treatments, reaching a maximum cell density of 13.06 × 10^5^ cells/mL, while specific growth rates were lower than those in the autotrophic control due to nutrient limitations or possible inhibitory compounds. Regarding nutrient removal, *D. salina* achieved 85.0% nitrate removal, 81.5% total ammonia nitrogen removal, and 46.3% nitrite removal in undiluted SAW. Additionally, the chemical oxygen demand was reduced by 80.3%, indicating substantial degradation of organic matter. In terms of biomass composition, lipid content increased significantly under SAW conditions, with a maximum of 67.1% dry weight in 25% diluted SAW, while the protein content decreased from 37.38% in GF2CM to 24.9–26.3% in the SAW treatments. The fatty acid profile remained favorable, with undiluted SAW cultures retaining a high proportion of polyunsaturated fatty acids, notably 12.0% α-linolenic acid. These findings support the feasibility of integrating *D. salina* in shrimp aquaculture effluent treatment systems, contributing simultaneously to environmental management and high-value-biomass generation. Future studies should explore scaling potential, continuous operation systems, and economic viability to consolidate *D. salina* as a component of circular aquaculture models.

## Figures and Tables

**Table 1:** Estimated parameters of the logistic model in terms of cell density growth of *Dunaliella salina* cultured in aquaculture wastewater.

Treatment	A	B	Xmid	Scal
0%	2.39	13.06 ^b^	2.81 ^b^	0.40 ^b^
25%	2.39	10.55 ^c^	2.70 ^b^	0.30 ^b^
50%	2.39	8.39 ^d^	2.68 ^b^	0.46 ^ab^
Reference (GF2CM)	2.39	99.50 ^a^	4.44 ^a^	0.60 ^a^

A is the asymptote on the left side; B is the asymptote on the right side; xmid is the time value of when the cell density is midway between the asymptotes; scal is the slope at the inflection point at the midrange. Different superscripts indicate significant differences (*p* < 0.05).

**Table 2:** Characterization of nutrients in aquaculture wastewater.

Variable									
Cultivation System	pH	Salinity (psu)	Total Nitrogen (mg/L)	Nitrate (mg/L)	Nitrite (mg/L)	Total Ammonia Nitrogen (mg/L)	Total Phosphorus (mg/L)	Chemical Oxygen Demand (mg O_2_/L)	Reference
Recirculation	7.28	0.26		40.67	5.52	5.32	8.82	96	[13]
Recirculation	5.22			52	<0.01	12.8	11.2	64.3	[49]
Recirculation			53.15	18.83		2.6	18.25	190	[50]
				125.5	28.7	443	5.8		[51]
BFT	8.11	29		0.83	0.12	0.25	0.5	49.11	[52]
BFT	8.4	34.07		1.3	4.2	1.3	1.2		[53]
BFT	7.74	37.23		0.3	0.3	0.7	2		[54]
BFT	7.7	10.3		0.38	0.73	2.54	0.51		[55]
BFT	7.4	36	3.1	1.7	0.6	0.8		458.1	This research

BFT, biofloc technology dominated by heterotrophic bacteria.

**Table 3:** The nutrient removal percentage of *Dunaliella salina* grown in aquaculture wastewater.

	Treatment			
Nutrient	Reference	0%	25%	50%
Nitrate	99.9 ± 0.0 ^a^	85.0 ± 2.9 ^b^	82.4 ± 4.2 ^b^	85.7 ± 1.9 ^b^
Nitrite	-	46.3 ± 3.1	48.1 ± 8.3	51.9 ± 2.8
TAN	45.3 ± 12.1 ^b^	81.5 ± 3.8 ^a^	75.9 ± 7.4 ^ab^	79.9 ± 4.2 ^a^
Total nitrogen	98.7 ± 0.1 ^a^	76.4 ± 1.4 ^b^	75.1 ± 4.5 ^b^	77.2 ± 1.4 ^b^
COD	59.6 ± 2.2 ^b^	80.3 ± 1.9 ^a^	82.1 ± 0.9 ^a^	79.4 ± 2.9 ^a^

TAN, total ammonia nitrogen; COD, chemical oxygen demand. Different superscripts indicate significant differences (*p* < 0.05).

**Table 4:** Biochemical characterization and pigment content of *Dunaliella salina* cultured in undiluted aquaculture wastewater (0% SAW), and in 25- and 50%-diluted SAW; the reference was grown in F2 Guillard medium.

	Treatment			
Biochemical	Reference	0%	25%	50%
Nutrient				
Protein (%)	37.38 ± 4.34 ^a^	24.9 ± 3.57 ^b^	24.67 ± 3.37 ^b^	26.33 ± 2.92 ^b^
Lipid (%)	19.44 ± 4.18 ^c^	26.59 ± 3.53 ^b^	67.1 ± 4.95 ^a^	58.81 ± 9.42 ^a^
Pigment				
Chl *a* (µg/mL)	13.07 ± 0.98 ^a^	7.00 ± 1.22 ^b^	7.78 ± 0.74 ^b^	5.3 ± 0.25 ^b^
Chl *b* (µg/mL)	3.19 ± 0.55	1.84 ± 0.15	2.88 ± 0.32	2.18 ± 0.37
Total carotenoid (µg/mL)	9.27 ± 0.21 ^a^	5.71 ± 1.02 ^b^	5.62 ± 0.53 ^b^	4.05 ± 0.22 ^b^

Different superscripts indicate significant differences (*p* < 0.05).

**Table 5:** Antioxidant enzyme of *Dunaliella salina* cultured in undiluted aquaculture wastewater (0% SAW), and in 25- and 50%-diluted SAW; the reference was grown in Guillard F2 culture medium.

	Treatment			
	Reference	0%	25%	50%
SOD (U/mg protein)	8.34 ± 1.24	5.07 ± 0.64	6.07 ± 0.9	5.67 ± 1.53
GPx (µM NADPH/min/mg protein)	0.16 ± 0.01	0.17 ± 0.06	0.2 ± 0.03	0.11 ± 0.01
CAT (U/mg protein)	0.89 ± 0.21	1.33 ± 0.56	1.69 ± 0.39	0.85 ± 0.15
APX (nM ascorbic acid/mg protein/min)	0.15 ± 0.01 ^a^	0.03 ± 0.01 ^b^	0.03 ± 0.01 ^b^	0.03 ± 0.00 ^b^

SOD, superoxide dismutase; GPx, glutathione peroxidase; CAT, catalase; APX, ascorbate peroxidase. Different superscripts indicate significant differences (*p* < 0.05).

**Table 6:** Fatty acid composition (% of total fatty acids) of *Dunaliella salina* cultured in undiluted aquaculture wastewater (0% SAW), and in 25- and 50%-diluted SAW; the reference was grown in Guillard F2 culture medium.

	Treatment			
Fatty Acid	Reference	0%	25%	50%
SFA				
C8:0	1.17 ± 0.25	1.57 ± 0.04	ND	ND
C12:0	1.17 ± 0.26	1.58 ± 0.04	ND	ND
C14:0	1.31 ± 0.28 ^b^	1.77 ± 0.05 ^b^	2.11 ± 0.23 ^ab^	2.72 ± 0.04 ^a^
C15:0	1.13 ± 0.28 ^b^	1.57 ± 0.05 ^b^	ND	2.54 ± 0.03 ^a^
C16:0	19.19 ± 0.86	18.07 ± 0.58	20.94 ± 1.46	19.21 ± 0.58
C17:0	1.23 ± 0.25 ^b^	1.59 ± 0.05 ^b^	1.92 ± 0.24 ^ab^	2.59 ± 0 ^a^
C18:0	1.95 ± 0.21 ^c^	2.36 ± 0.05 ^bc^	2.74 ± 0.25 ^ab^	3.23 ± 0.06 ^a^
C20:0	1.98 ± 0.1 ^b^	2.06 ± 0.04 ^b^	2.48 ± 0.23 ^ab^	2.95 ± 0.01 ^a^
C22:0	2.48 ± 0.18 ^ab^	2.31 ± 0.09 ^b^	2.76 ± 0.23 ^ab^	3.04 ± 0 ^a^
ΣSFA	31.61 ± 0.76 ^b^	32.88 ± 0.57 ^b^	32.95 ± 0.29 ^b^	36.27 ± 0.47 ^a^
MUFA				
C14:1	1.32 ± 0.24 ^b^	1.79 ± 0.15 ^b^	2.05 ± 0.23 ^ab^	2.67 ± 0.01 ^a^
C15:1	1.18 ± 0.26	1.59 ± 0.06	ND	ND
C16:1	1.83 ± 0.05 ^b^	1.79 ± 0.04 ^b^	2.14 ± 0.24 ^b^	2.81 ± 0.02 ^a^
C17:1	3.02 ± 0.86	2.36 ± 0.06	2.65 ± 0.24	3.00 ± 0.07
C18:1n9	18.4 ± 0.56	17.63 ± 0.29	18.32 ± 1.08	16.05 ± 0.3
C20:1n9	1.36 ± 0.17 ^c^	1.63 ± 0.05 ^bc^	2.14 ± 0.17 ^ab^	2.69 ± 0.05 ^a^
ΣMUFA	27.11 ± 0.79	26.79 ± 0.17	27.3 ± 0.29	27.21 ± 0.31
PUFA				
C18:2n6	3.34 ± 0.13	7.51 ± 3.95	4.15 ± 0.15	4.39 ± 0.04
C18:3n6	16.11 ± 1.23	10.16 ± 4.26	15.79 ± 0.66	12.95 ± 0.24
C18:3n3	14.14 ± 1.49 ^a^	12.02 ± 0.36 ^ab^	12.71 ± 0.21 ^ab^	9.77 ± 0.48 ^b^
C20:2n6	1.16 ± 0.27	1.6 ± 0.05	ND	ND
C20:3n3	1.15 ± 0.26	1.58 ± 0.05	ND	ND
C20:4n6	1.18 ± 0.26	1.58 ± 0.05	ND	ND
ΣPUFA	37.09 ± 1.95 ^a^	34.45 ± 0.65 ^a^	32.64 ± 0.53 ^a^	27.1 ± 0.67 ^b^
Σn3	15.3 ± 1.24 ^a^	13.6 ± 0.33 ^a^	12.71 ± 0.21 ^ab^	9.77 ± 0.48 ^b^
Σn6	20.63 ± 0.96 ^a^	19.25 ± 0.39 ^ab^	19.94 ± 0.52 ^ab^	17.34 ± 0.19 ^b^
Σn3/Σn6	0.74 ± 0.03 ^a^	0.71 ± 0.01 ^ab^	0.64 ± 0.02 ^bc^	0.56 ± 0.02 ^c^

ND, not detectable; SFAs, saturated fatty acids; MUFAs, monounsaturated fatty acids; PUFAs, polyunsaturated fatty acids. Different superscripts indicate significant differences (*p* < 0.05).

## Data Availability

The original contributions presented in this study are included in the article. Further inquiries can be directed to the corresponding author.
